# Photophysiological cycles in Arctic krill are entrained by weak midday twilight during the Polar Night

**DOI:** 10.1371/journal.pbio.3001413

**Published:** 2021-10-19

**Authors:** Jonathan H. Cohen, Kim S. Last, Corie L. Charpentier, Finlo Cottier, Malin Daase, Laura Hobbs, Geir Johnsen, Jørgen Berge

**Affiliations:** 1 School of Marine Science & Policy, University of Delaware, Lewes, Delaware, United States of America; 2 Scottish Association for Marine Science, Oban, United Kingdom; 3 Department of Biology, Stetson University, DeLand, Florida, United States of America; 4 UiT, The Arctic University of Norway, Faculty for Biosciences, Fisheries and Economics, Department for Arctic and Marine Biology, Tromsø, Norway; 5 Department of Mathematics and Statistics, University of Strathclyde, Glasgow, United Kingdom; 6 University Centre in Svalbard, Longyearbyen, Norway; 7 Centre of Autonomous Marine Operations and Systems, Department of Biology, Norwegian University of Science and Technology, Trondheim, Norway; Lund University, SWEDEN

## Abstract

Light plays a fundamental role in the ecology of organisms in nearly all habitats on Earth and is central for processes such as vision and the entrainment of the circadian clock. The poles represent extreme light regimes with an annual light cycle including periods of Midnight Sun and Polar Night. The Arctic Ocean extends to the North Pole, and marine light extremes reach their maximum extent in this habitat. During the Polar Night, traditional definitions of day and night and seasonal photoperiod become irrelevant since there are only “twilight” periods defined by the sun’s elevation below the horizon at midday; we term this “midday twilight.” Here, we characterize light across a latitudinal gradient (76.5° N to 81° N) during Polar Night in January. Our light measurements demonstrate that the classical solar diel light cycle dominant at lower latitudes is modulated during Arctic Polar Night by lunar and auroral components. We therefore question whether this particular ambient light environment is relevant to behavioral and visual processes. We reveal from acoustic field observations that the zooplankton community is undergoing diel vertical migration (DVM) behavior. Furthermore, using electroretinogram (ERG) recording under constant darkness, we show that the main migratory species, Arctic krill (*Thysanoessa inermis*) show endogenous increases in visual sensitivity during the subjective night. This change in sensitivity is comparable to that under exogenous dim light acclimations, although differences in speed of vision suggest separate mechanisms. We conclude that the extremely weak midday twilight experienced by krill at high latitudes during the darkest parts of the year has physiological and ecological relevance.

## Abstract 2

This study shows that ambient light cycles set an internal rhythm that controls visual sensitivity of Arctic krill during the Polar Night – the darkest part of the year, when the sun remains below the horizon all day. This demonstrates that biologically relevant photoperiods can be achieved during this time of "midday twilight".

## Introduction

Light affects nearly all biological activities such as finding food, avoiding predators, migration, and mate selection and is central to driving visual processes. Since the light available to an organism varies with behavior, habitat, and season, a myriad of photoreceptors have evolved, which are able to operate, often in the same eyes, over changes in illumination (irradiance) by more than a factor of 10^11^ [1,2]. Coupled to this are changes in the spectral quality of light further dependent on elevation of the sun or moon in the sky both above and below the horizon. For example, at twilight with the sun below the horizon, a relative increase at blue and red wavelengths emerges as yellow wavelengths are selectively absorbed by atmospheric ozone, a phenomenon termed the “Chappuis Effect” [3,4]. Light in the sunlit shallow epipelagic (0 to 200 m) aquatic domain can be even more complex with even relatively small changes in an organisms’ vertical position resulting in very large changes in irradiance and spectral composition of light. For example, light intensity decreases by approximately 1.5 log units for every 100 m in clear ocean water, narrowing from full spectrum to the blue region [5].

Vision and light detection have evolved in parallel with the circadian clock, a self-sustained molecular machine reliant on positive and negative autoregulatory feedback loops and synchronized to external environmental cycles (most commonly light) via entrainment [6,7]. In marine organisms, other entrainment cues (e.g., tides, temperature, and salinity) are also often implicated [8]. Of particular significance is that the clock relies on the day–night cycle for temporally structuring both short-term processes (e.g., daily foraging) and long-term processes (e.g., gametogenesis) via photoperiodism [9]. Importantly, spectral cues characteristic of twilight can provide sufficient information to entrain circadian clocks [10,11]. For example, the blue dominant solar spectrum characteristic at twilight is consistent with the spectral absorption of cryptochromes known to be widely involved in circadian entrainment [12–14]. Light, therefore, plays multiple roles, among them vision and circadian clock entrainment, which, in combination, provide highly evolved and accurate spatial and temporal information for most behavioral and physiological processes.

**Fig 1:**
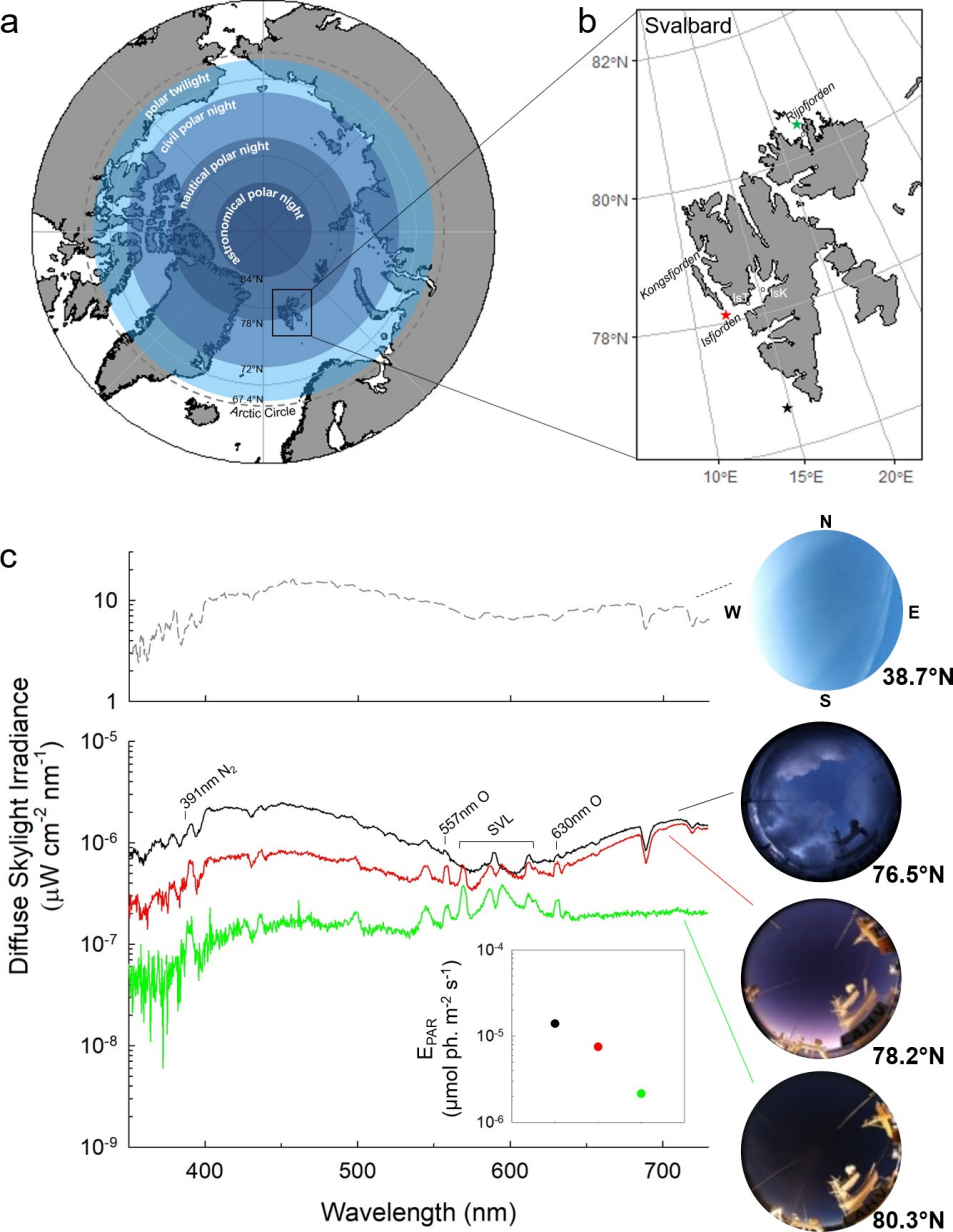
Atmospheric light at solar noon across latitudes during the Polar Night. **(a)** Northern Hemisphere of Earth, centered on the North Pole. Concentric circles represent the increasing extent of Polar Night as latitude increases; bands are based on the sun’s elevation relative to the horizon at solar noon on the winter solstice: civil twilight (0 to 6°), nautical twilight (6 to 12°), and astronomical twilight (12 to 18°) (modified from [78]). **(b)** Svalbard archipelago, with locations of light measurements shown as stars, and locations of krill collection for physiological experiments (Fig 3, S2 Fig) shown as open circles. Basemap for panel (a) is from Natural Earth Data using the combined datasets for Physical Vectors with Land (https://naturalearth.s3.amazonaws.com/10m_physical/ne_10m_land.zip) and Minor Islands (https://naturalearth.s3.amazonaws.com/10m_physical/ne_10m_minor_islands.zip). Basemap for panel (b) is from Geonorge using S100 Map data (https://kartkatalog.geonorge.no/metadata/s100-map-data/bd6050e8-7182-459b-9989-66c4ecbae874). **(c)** Hyperspectral irradiance measured at sunset for a midlatitude location (38.7^o^N at 21:54 GMT, upper panel) and 3 high-latitude locations around Svalbard in January (lower panel). For high-latitude spectra, diagnostic spectral bands from aurora borealis (391, 557, and 630 nm) and the research vessel’s SVLs are annotated. All-sky images taken at the time of spectrum acquisition are shown at right. Inset is irradiance integrated as photosynthetically active radiation (400 to 700nm, E_PAR_). Black, red, and green colors represent 3 stations of increasing latitude (measured at times: 11:35, 11:11, and 11:50 GMT, respectively), which correspond to stars (panel b). For data, see S1 Data. SVL, sodium vapor lamp.

Organisms inhabiting high latitudes experience a greater annual photoperiodic range than those at lower latitudes. At the poles, the sun is either permanently above or below the horizon for large parts of the year (Midnight Sun and Polar Night, respectively). During the Polar Night, traditional definitions of day and night and seasonal photoperiod become irrelevant since there are only “twilight” periods. These are defined by the sun’s elevation below the horizon at midday [4] and therefore considered hereafter as simply “midday twilight” (Fig 1A). Observations suggest biological relevance of midday twilight. Indirect acoustic measurements in Svalbard fjords during the Polar Night have revealed diel vertical migration (DVM) of zooplankton [15–17] and lunar vertical migrations (LVMs), which are also reflected across the Arctic Ocean as a whole [18,19]. However, direct observations on Arctic zooplankton endogenous visual sensitivity during the Polar Night are lacking [20], with the assumption that diel cycling of midday twilight is insufficient to entrain endogenous rhythms at this time of year [21]. With increased attention on poleward range shifts under climate change in marine organisms [22], it is now recognized that understanding photic barriers to such migrations is critical [23,24].

In order to understand the role of light in vision and endogenous rhythms during Polar Night, we ask whether krill show rhythmic visual sensitivity during Arctic midday twilight. We used an in situ observational and experimental approach to study boreal/Arctic krill (*Thysanoessa inermis*), which dominate the Arctic macrozooplankton community and undergo extensive DVM [25]. The life cycle of krill is influenced by photoperiod, which determines annual patterns of metabolic activity, sexual maturity, and lipid utilization [26]. Furthermore, much like in Antarctic krill (*Euphausia superba*) [20,27], it is hypothesized that Arctic krill also possess a functional circadian clock. The aim of this paper is to (a) understand the components of atmospheric light which make up midday twilight during Arctic Polar Night; (b) observe the natural Arctic zooplankton community to characterize DVM at this time; (c) determine spontaneous changes in krill visual sensitivity under constant lab conditions; and (d) contextualize these endogenous changes in visual sensitivity with dim light acclimations. In order to provide a quantitative measure of Polar Night midday twilight, point sample hyperspectral light measurements were taken from a research vessel spanning a range of latitudes in the Barents Sea and Arctic Ocean. In addition, and to gain an understanding of daily changes in irradiance over time when the light cycle is driven by midday twilight, light data were gathered from a land-based light observatory [4]. Biological responses to changes in twilight cycles were determined acoustically near the light observatory. Finally, 3 types of electrophysiological visual sensitivity experiments were conducted on *T*. *inermis*: The first tested endogenous sensitivity rhythms over time in an individual, while the second compared visual responses in multiple krill across irradiance levels tested during subjective midday and midnight periods. Finally, the third experiment was used to contextualize endogenous visual sensitivity changes with those experienced under simulated ambient midday twilight intensities.

## Results and discussion

### Polar Night is a time of darkness punctuated by midday twilight

Using a spectroradiometer optimized for dim light detection [28], we quantified spectral irradiance of diffuse skylight at solar noon during the Polar Night from 76.5° N to 81° N under a range of weather conditions from a research vessel platform (Fig 1). Broad peaks are present at blue and red wavelengths, with ozone absorption in the yellows (approximately 600 nm, Chappuis band) (e.g., [29]). Artificial light from the ship’s sodium vapor lighting is evident as peaks at approximately 590 nm (Fig 1C), but other peaks in this range represent ambient light (e.g., aurora borealis: 557 nm and 630 nm). Integrated diffuse skylight irradiance measured at the water’s surface (400 to 700 nm, E_PAR_) ranged from 2.2 × 10^−6^ to 2.2 × 10^−5^ μmol photons m^−2^ s^−1^ (Fig 1C, inset). These measurements demonstrate that atmospheric light at midday during Polar Night is, as expected, characteristic of twilight spectral composition at lower latitudes (e.g., [30]) (Fig 1C, upper panel).

In order to capture the largest change in irradiance over the diel cycle, we measured a time series of diffuse skylight irradiance during Polar Night north of Rijpfjorden, Svalbard (80° 37.79N 22° 4.14E) over the midday period. During this time, the sun remained below the horizon reaching a maximum elevation of −11.69°, while the moon (approximately 89% illuminated) remained above the horizon but descended from 5.6° to 0.3° elevation (S1 Fig). A classic solar-driven diel light cycle (e.g., [31]) is damped at these latitudes during the extreme photic conditions of Polar Night. While a sun-dominated photoperiod following solar elevation remains evident (S1A and S1B Fig), moonlight and green/red light from the aurora borealis serve to extend what would be an otherwise much shorter solar-driven diel light cycle (S1C and S1D Fig).

### Oscillating Polar Night light intensity is consistent with cyclic behaviors in marine zooplankton

**Fig 2:**
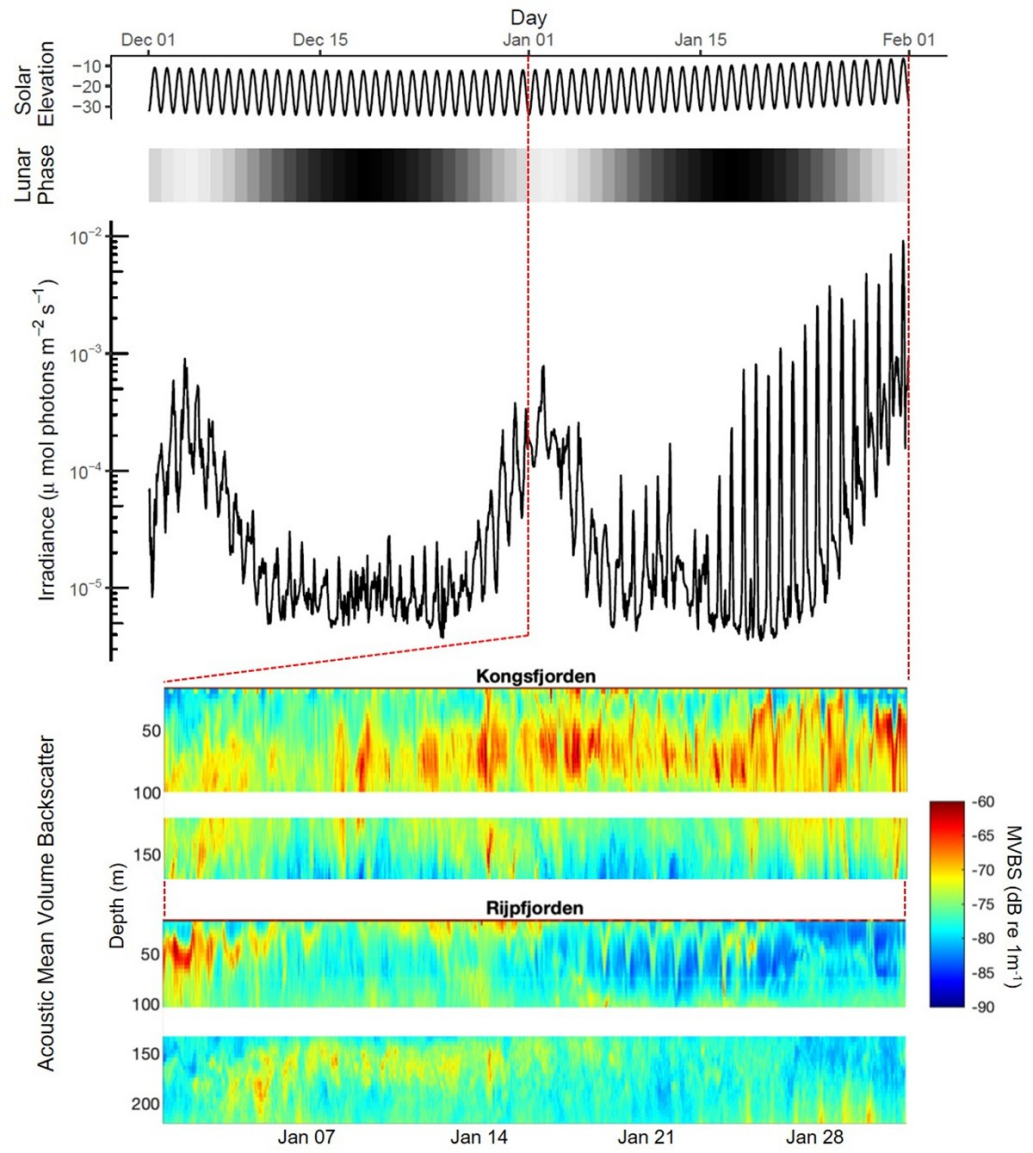
Cyclic atmospheric light and hydroacoustic patterns during Polar Night. Solar elevation (degrees relative to horizon) at Ny-Ålesund, Svalbard (Kongsfjorden) during Polar Night, December 2017 to February 2018. Grayscale bar represents daily moon fullness (black = 0%, new moon; light gray = 100%, full moon). Atmospheric irradiance (E_PAR_) at Ny-Ålesund is replotted from [4]. Red dashed vertical lines denote the month of January 2018, during which hydroacoustic observations were conducted with ADCPs. Acoustic MVBS (dB re 1 m^−1^) detection of zooplankton in the water column is plotted during January for Kongsfjorden and Rijpfjorden (Svalbard). Period analysis (S1 Table) shows significant diel rhythmicity at all depths and throughout January. Missing acoustic data between approximately 100 and 130 m is due to a “blind zone” of upward/downward facing ADCPs. For data, see S1 Data. We see evidence at the community level that the cyclic light we measured during January 2018 influences in situ migration behavior of marine zooplankton. We examined acoustic backscatter throughout a water column of approximately 200 m in Kongsfjorden and Rijpfjorden to monitor cyclic changes of zooplankton biomass over time and with depth (Fig 2, dashed red box). These biomass changes reflect both DVM and LVM of zooplankton [15,18,32–34], which is commonly triggered by the ambient light cycle [35,36]. Period analysis of acoustic backscatter revealed significant migrations at both stations in January when the maximum solar elevation at midday was only −6.2° (S1 Table). Significant periods in the circadian range for biomass movement were detected throughout the month of January in Kongsfjorden (57% of depth bins) and in Rijpfjorden (61% of depth bins). These data agree with previous observations (e.g., [15]) showing migrations that continue during the Polar Night in January are driven by solar and lunar cycles. While we do not have net samples from the acoustic mooring location to confirm the identities of the zooplankton migrators, previous net sampling coincident with acoustic surveys in these fjords during Polar Night show that krill (*Thysanoessa* spp.) are the dominant migrators and contribute >90% of macrozooplankton biomass [37,38]. Our own net sampling at this time of year further confirms this observation. ADCP, acoustic doppler current profiler; MVBS, mean volume backscatter.

Using atmospheric light data collected during the darkest part of Polar Night in Ny-Ålesund, Svalbard (78.9°N, 11.9°E) [4], we determined weekly periods (Tau, T) in light intensity (E_PAR_) across the lunar cycle during the month of January 2018 (Fig 2). These data reveal overt diel light intensity changes, onto which the monthly and daily lunar light cycles are superimposed. Lomb–Scargle period estimates for January were T = 24.8 hours during the first week (full moon) and T = 24.0 hours for all subsequent weeks. Increasing diel light intensities as a consequence of solar elevation at the end of January mask the rise and fall of the subsequent full moon. Regardless, light intensity remains cyclic with lunar and solar day periods, throughout midday twilight during the Polar Night.

### Krill express nocturnal endogenous rhythms in visual sensitivity

We subsequently tested *T*. *inermis* in January from 3 Svalbard fjord locations (Rijpfjorden, Kongsfjorden, and Isfjorden) (Fig 1B) for rhythmic visual sensitivity change by extracellular electroretinogram (ERG) recording (e.g., [28,39]). The choice of *T*. *inermis* was based on its dominance among *Thysanoessa* species within Svalbard fjords, including the fjord locations we sampled [40,41].

We observed a significant rhythm in visual sensitivity in ERG recordings from the eye of an individual tested by repeated stimulation with dim light flashes at 1°C (S2A Fig). ERG amplitude showed a period of 20.4 hours, with peaks in visual sensitivity in phase with low solar elevation (i.e., subjective night) (S2B Fig). Several studies support endogenous circadian rhythms in krill, including Antarctic krill *E*. *superba* [42,43] and northern krill *Meganyctiphanes norvegica* [44]. While these ERG time series data suggest that comparable endogenous rhythmic processes may occur in *T*. *inermis*, we adopted a different experimental approach to validate that conclusion. This was done to maximize replication during the limited ship time available in conducting these physiological experiments on live, freshly collected animals.

**Fig 3:**
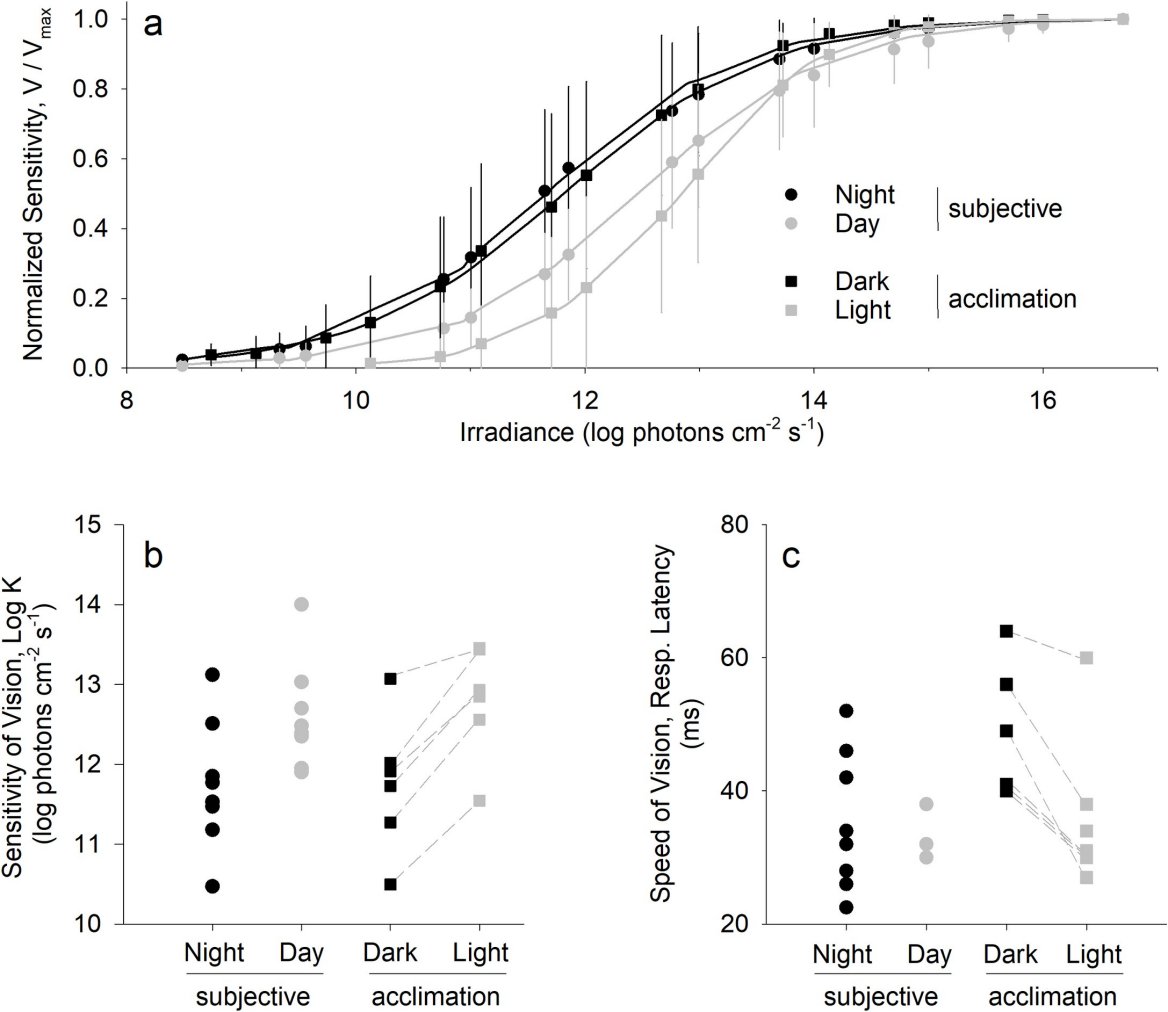
Endogenous and exogenous change in krill visual sensitivity and speed with midday twilight. **(a)** Response irradiance (V-log*I*) curves for *T*. *inermis*, with experiments testing endogenous (circles, subjective) and exogenous (squares, acclimation) sensitivity change at 1°C. Endogenous experiments were conducted during subjective night (black circles, *n* = 8) and day (gray circles, *n* = 8). Exogenous experiments were conducted during the day with krill (*n* = 6) tested first under dark acclimation (black squares) and then under simulated midday twilight acclimation (gray squares). Symbols represent means (± 1 SD) of V-log*I* models fit to normalized ERG data, with aggregate data fit by a V-log*I* model (solid lines). **(b)** Log irradiance at half-saturation for V-log*I* models (Log K) of each individual krill, with symbols as described for (a). Light acclimation experiments were paired Dark-Light, so points corresponding to the same individual are connected by a dashed line. Log K was lower during both subjective night (*p* = 0.038, rank sum test) and dark acclimation (*p* = 0.031, signed rank test) when compared to corresponding subjective day and light acclimation treatments in each experiment, respectively. **(c)** Speed of vision as determined by response latency (elapsed time between onset of the light flash and onset of the photoreceptor response at 50% V_max_). Response latency was analyzed for all individuals plotted in (b) and plotted with the same symbol designation. Response latency did not differ between subjective night and day (*p* = 0.833, rank sum test), but decreased with light acclimation (*p* = 0.031, signed rank test). For data, see S2 Data.

For these further experiments, we tested whether diel visual sensitivity rhythms were endogenous by determining the half-saturation (Log K) values from response irradiance (*V*-log*I*) curves for *T*. *inermis* eyes. The *V*-log*I* approach has been used in previous studies to show a difference in visual sensitivity over the diel cycle consistent with changes in ERG amplitude [39,45–47]. We conducted measurements around the times of subjective midday and midnight periods (*n* = 8 individuals each) (Fig 3A). Freshly collected krill were held at 1°C in darkness for 24 hours before experiments began, with all diel environmental cycles removed to ensure that animals were in a free-running state. *V*-log*I* curves support endogenous control of visual sensitivity as Log K values were lower during subjective midnight than during subjective midday, but visual speed as response latency remained unchanged (Fig 3B and 3C). We can therefore assume that ambient light levels and extremely short duration midday twilight experienced by krill in situ during the Polar Night are sufficient to entrain a circadian rhythm of visual sensitivity. In further laboratory experiments (*n* = 6 individuals), we reveal comparable visual sensitivity changes for krill under simulated midday twilight acclimation (Fig 3A and 3B). Visual speed increased with light acclimation, demonstrated as a decrease in response latency, which contrasts with results from our free-running experiments (Fig 3C). Thus, different mechanisms are likely involved in observed diel endogenous sensitivity changes as compared to the exogenous sensitivity changes observed here and for other polar crustaceans [48–50].

### Ecological benefits of extreme light sensitivity in krill

**Fig 4:**
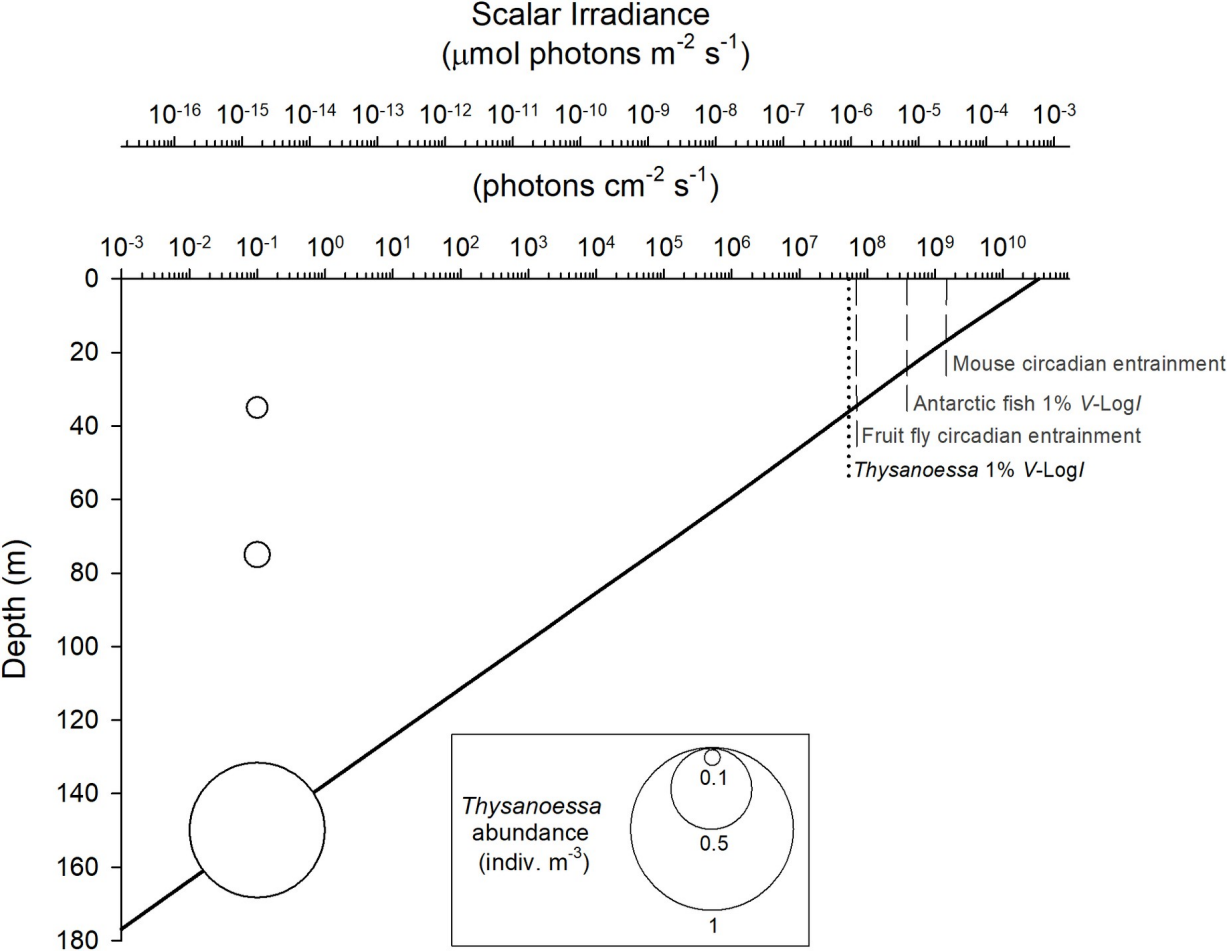
Modeled underwater light during Polar Night. Underwater light intensity plotted as scalar irradiance (E_o_; irradiance on a point from all directions weighted equally). Light values were derived from radiative transfer model results of diffuse skylight irradiance measured at Rijpfjorden, Svalbard (80° 37.79N 22° 4.14E) at solar noon on January 15, 2016, then propagated through the water column (bold black line showing irradiance as a function of depth). The dotted vertical line denotes the visual sensitivity threshold for *T*. *inermis* (1% of V_max_ for subjective midnight *V-*log*I*). Dashed vertical lines show thresholds for light-mediated processes in a range of other taxa: entrainment of circadian rhythms in fruit flies [53] and mice [55]; 1% of V_max_ from *V-*log*I* for Antarctic fish *Pagothenia* [56]. Depth-stratified abundance of *T*. *inermis* at Rijpforden (06:30 on January 14, 2016) is plotted as bubbles of proportional size. For data, see S2 Data.

The light-mediated rhythmic behavioral and physiological processes we have demonstrated occur at extremely low intensities of solar/lunar illumination. The atmospheric irradiance values we measured (Figs 1C and 2) are conservative in representing the underwater light field as they do not account for refraction of light at the water surface (approximately 4% of incoming E_PAR_) and subsequent light attenuation with depth [51]. To determine biologically relevant underwater light, we used our atmospheric measurements in radiative transfer models to quantify the underwater light krill would experience in the water column at 81° N during Polar Night sampling in January (Fig 4). Intensities sufficient to evoke visual responses in krill extended to over 40 m below the surface, encompassing thresholds for photoentrainment in model crepuscular/nocturnal terrestrial organisms [52–55] and visual sensitivity thresholds in polar fish (i.e., 1% of *V*-Log*I* curve [56]). As photosensitivity may be nonvisual or extraocular [10], it is likely that light intensities sufficient for entrainment of cyclic processes, such as ERG rhythms in krill, are even less than that required for visual photoreception. The vertical distribution of *T*. *inermis* in Rijpfjorden during the morning (approximately 06:30 UTC, 07:30 local) suggests that krill are mostly at their daytime depths (consistent with Fig 2), which exceeds their visual threshold (Fig 4).

Endogenous clocks that underpin physiological and behavioral changes are primarily adaptive in their anticipatory capacity in environments where entrainment cues may, or may not, be sufficient [57–59]. Our ERG data show endogenous, rhythmic increases in visual sensitivity for krill during the subjective night and a reduction during the subjective day (Fig 3, S2 Fig), consistent with the nighttime diel migrations of krill (Fig 2). Circadian cycles of visual sensitivity mediated by retinal/interneuron processes and/or screening pigment migrations are common among terrestrial and aquatic animals (e.g., [60–62]). In a particularly well-studied marine organism, the horseshoe crab *Limulus polyphemus*, lateral compound eyes undergo changes in structure, gene expression, and rhabdom biochemistry to increase visual sensitivity at night (reviewed in [63,64]). This sensitivity increase is understood to be adaptive in nighttime mating at all states of the tide, day or night [65]. Equally for krill, this increased nighttime visual sensitivity could enhance the effectiveness of its bioluminescence. Krill perform counter-illumination by detecting downwelling light and matching the intensity with light emitted from photophores on their ventral surface [66–68]. This casts a light shadow, visually masking them from predators below [69,70]. Additionally, krill may feed by detecting bioluminescent flashes of prey [71]. Endogenous control of either would be adaptive when environmental light signals are weak or variable. Whether circadian rhythms in visual sensitivity persist during periods of constant light (i.e., Midnight Sun) remains an open question.

The most striking feature of meso- and macrozooplankton biology is arguably their vertical migrations. Typically, this behavior is understood to be a balance between predator avoidance at depth during the day and feeding near the surface at night under the cover of darkness (reviewed in [36]). Increased visual sensitivity at night may strengthen the swimming response to changes in light at a time when these animals are migrating (Fig 2). Suppressed visual sensitivity during the day may, in turn, minimize responses to episodic changes in light (e.g., passing clouds) that would otherwise evoke vertical swimming, reducing metabolic costs and risk from visual predators. Even though the Polar Night presents little food from primary production, heterotrophic predators may still rely on migration to find their prey (e.g., [37]). With an endogenous rhythm in visual sensitivity, krill would anticipate the time of night through circadian entrainment and therefore return to the surface to feed omnivorously. This may explain the significant migrations we observed at depth (>100 m) (e.g., S1 Table and Fig 2). Previous molecular and behavioral studies have come to a similar conclusion for vertical migrators (e.g., [72–74]).

We conclude that midday twilight during high Arctic Polar Night has physiological and ecological relevance even at less than 1-fold change in diel light intensity, as compared to a 7-fold change at these locations during the equinoxes (e.g., [4]). It appears that in krill at least, circadian rhythms may be entrained by nonclassical midday twilight with required irradiance levels among the lowest for any organism to date. While mechanisms for this process are currently unknown, as are the potential role for other entrainment cues and pathways [74–77], these data provide a window into the likely flexibility of visual physiology in organisms from other dim light habitats.

## Materials and methods

To analyze periodicity in light cycles during the darkest portion of the Polar Night (December/January), we used data from an atmospheric irradiance (E_PAR_) time series measured near Ny-Ålesund, Svalbard (78.9°N, 11.9°E) in close proximity (<5 km) to where the Kongsfjorden acoustic data were collected (Fig 2). These measurements were taken with an all-sky camera-based light sensor (Canon EOS 5D Mark III with 8 mm fisheye lens, Melville, New York, USA) calibrated for E_PAR_ [4,79]. Period estimates were determined at weekly intervals for the irradiance time series using Lomb–Scargle periodograms within the Time Series Analysis (TSA) Serial Cosinor 6.3 package (S1 Table). Hyperspectral irradiance spectra of diffuse skylight during the winter Polar Night period were taken in January 2016 from the observation deck above the bridge of the R/V Helmer Hanssen using an Ocean Optics QE Pro spectroradiometer with Spectralon reflectance plate having a detection limit of approximately 4 × 10^−10^ μmol photons m^−2^ s^−1^ (see [28,80] for detailed methods). Artificial light was minimized by extinguishing vessel lights on this part of the ship, but some work lights aft and below the observation deck remained illuminated for operational safety reasons; the spectral quality of these deck lights was measured, and its minimal influence readily differentiated from environmental sources in diffuse skylight spectra (see Fig 1C). Point measurements were made at solar noon on January 10 and 12 at locations south and west of Svalbard (generally clear skies, some clouds on 76.5°N may have attenuated light) and on January 15 north of Svalbard over an extended 3-hour period centered on solar noon (Fig 1, S1 Fig). The sun’s elevation relative to the horizon at solar noon ranged between −8.1° (i.e., nautical twilight) and −12.5° (i.e., astronomical twilight) (see [4] for further discussion of twilight definitions). Coincident with these hyperspectral irradiance measurements, an all-sky camera (as described above) adjacent to the spectroradiometer was used to capture aligned images of atmospheric conditions (e.g., clouds, sun/moon position, and aurora activity), with camera settings to a constant ISO of 12800 (light sensitivity), aperture (f) of 4.5, white balance manually set to “daylight,” and using the shutter speed as the only variable [28,79]. For comparison of the spectral composition of midday twilight, hyperspectral irradiance and an all-sky image were measured at twilight (after sunset) in January 2021 at a midlatitude location (Lewes, Delaware, USA; 38.7°N, 75.1°W).

Acoustics have been used in identifying vertical migration in zooplankton with calibrated echosounders [15,32–34]. In order to determine DVMs of the zooplankton community in the natural light environment, we used 2 RDI Workhorse 300 kHz acoustic doppler current profilers (ADCPs) moored in Kongsfjorden (78°58′N, 11°47′E) and Rijpforden (80°13′N, 22°26′E) in January 2018. All ADCP data were checked for quality using the RDI correlation index (a measure of signal to noise ratio) recorded at the instrument. Acoustic volume backscattering strength (S_v_; dB re 1 m^−1^) was derived from echo intensity following the method described in [81] and later employed by [15,33,82,83] (Fig 2).

We investigated visual sensitivity over the diel cycle in an individual krill using a shipboard assay (S2 Fig). Krill (*T*. *inermis*) were collected using a midwater trawl net from the upper 200 m in Kongsfjorden (78°57′N, 11°57′E) on January 20, 2016. Immediately upon recovery of the net, the cod end was transferred to a light-tight bucket and brought to a 3°C cold room aboard the vessel (ambient water temperature approximately 2°C). Once there, animals were sorted under dim red light, and a krill was immediately prepared for an experiment. In order to determine visual sensitivity over time, we measured the magnitude of extracellular ERGs in response to a standardized 50-ms flash of 488-nm light at 3.65 × 10^−9^ photons cm^−2^ s^−1^, given at 15-minute intervals with the animal otherwise in darkness. Equipment was as described in detail elsewhere [28,39]. Briefly, under dim red light (Schott RG630 longpass filter), *T*. *inermis* were restrained and submerged in a temperature-controlled seawater bath within a light-tight Faraday cage. An epoxy-insulated tungsten microelectrode (125-μm shank, FHC, Bowdoin, Maine, USA) was positioned subcorneally in the eye, and a reference electrode was placed in the seawater bath. Seawater bath temperature was maintained at 1°C as measured by a thermocouple positioned at the krill eye. The animal was not fed for the duration of the experiment. Periodicity in visual sensitivity was calculated using the Lomb–Scargle periodogram with results suggesting significant rhythmicity (S2B Fig). In order to maximize replication, we adopted the response irradiance (*V*-log*I*) experimental design described below focusing purely on sensitivity during subjective twilight day and twilight night periods.

To confirm the rhythmic visual sensitivity results and demonstrate that *T*. *inermis* collected at different latitudes possess endogenous rhythms entrained under twilight cycles, we determined *V*-log*I* curves by ERG recording (Fig 3). Krill were collected by midwater trawl from Isfjorden-Karlskronadjupet (IsK; 78.32°N, 15.17°E on January 11, 2016 at 06:10 UTC), Rijpfjorden (80.30°N, 22.27°E on January 14, 2016 at 09:49 UTC), and Isfjorden-Trygghamna (IsT; 78.23°N, 13.83°E on January 17, 2016 at 16:37 UTC) (Fig 1B). Netted animals were treated as described above, only this time held in darkness for at least 24 hours without supplemental food until used in ERG experiments. Given this constant dark acclimation and removal of diel zeitgebers, any rhythmic activity present in the physiological experiments described below is considered to be endogenous [84]. We ceased testing krill 72 hours after collection. We quantified visual sensitivity by ERGs using *V*-log*I* curves during the subjective twilight day and twilight night periods, defined here as 09:00 to 15:00 and 21:00 to 03:00, respectively. Equipment and experimental details were as described above and in detail elsewhere [28,39]. The light stimulus was set to the wavelength of maximum spectral sensitivity for *T*. *inermis* (490 ± 7 nm, full width at half maximum) [28]. *V*-log*I* curves were generated for individual krill during the times of subjective twilight day (*n* = 8) and twilight night (*n* = 8) periods once ERG magnitude in response to a periodic dim test flash remained constant for a period of 1 hour. Different individuals were used for each replicate, with electrode position and depth in the eye kept constant across individuals to minimize variability in ERG response [39]. Peak-to-peak response heights of the ERG waveform (*V*) were measured over a range of 100-ms light intensity flashes (log irradiance; log *I*) spanning several orders of magnitude. We modeled *V*-log*I* data to determine the log irradiance evoking 50% of the maximum response amplitude (Log K) [85]. We estimated speed of vision (temporal resolution) for flashes at Log K by determining the response latency as the elapsed time between onset of the light flash and onset of the photoreceptor response [39]. We tested for differences in Log K and response latency between subjective twilight day and twilight night by rank sum tests.

We supplemented these experiments on free-running krill with other data on exogenous effects of light on visual sensitivity in *T*. *inermis*. For these experiments, we used krill collected from Kongsfjorden (78.961°N, 11.895°E at 17:21 UTC on January 15, 2015) employing the general electrophysiological protocols described above. Following collection, krill were maintained in constant darkness at 2.6°C (± 0.1 SD) within a 200-L flow-through tank fed by pumped Kongsfjorden water. Within 2 weeks of collection, we tested visual sensitivity (*V*-log*I*) and speed (response latency) at 1°C under dark and light acclimation treatments. In a given experiment, an individual krill (*n* = 6) was acclimated to darkness, defined by a constant ERG magnitude over a period of 1 hour in response to a dim test flash. After a *V*-log*I* curve was generated for the dark-acclimated krill, we light acclimated that individual to broadband blue light (Ocean Optics HL-2000 QTH lamp with Schott BG-18 broadband and 4.0 OD neutral density filters) yielding 5 × 10^−4^ μmol photons m^−2^ s^−1^ measured at the position of the krill eye. After a constant ERG magnitude was observed over a period of 1 hour in response to a dim test flash, a second *V*-log*I* curve was determined for this individual, which remained in a constant state of light acclimation as judged by dim test flashes throughout the experiment. We modeled *V*-log*I* data and calculated response latency as described above. Because dark and light acclimation were conducted sequentially in the same individuals, we compared Log K and response latency values between paired dark and light acclimation treatments with signed rank tests.

In order to relate light sensitivity of krill in their habitat to that of other species studied for dim light rhythmic processes, we used the radiative transfer model HydroLight 5.2 to characterize the underwater light field during midday twilight (Fig 4). Light input to the model was diffuse downwelling atmospheric spectral irradiance measured near Rijpfjorden during solar noon, with additional parameters and model details described in [28,86]. Model output was scalar spectral irradiance (E_o_) from 400 to 700 nm at 10-nm resolution.

To assess krill distributions relative to modeled underwater light, we sampled krill in a single vertically stratified net tow in Rijpfjorden on January 14, 2016 at 06:33 UTC at the approximate location of krill sampling for ERG experiments using a Hydro-Bios Multinet (0.25 m^2^) with mesh size 180 μm. Nets were lowered to 265 m and programmed to close 200 to 100 m, 100 to 50m, 50 to 20 m, 20 to 0 m. Cod end collections were fixed in 4% formaldehyde, and *T*. *inermis* abundances were later enumerated.

All work was carried out according to the Healthy, Safety and Environment guidelines of the local and national authorities for conducting fieldwork on Svalbard (see www.unis.no), and the project was entered into the Research in Svalbard (RiS) database with project number 10624 (https://www.researchinsvalbard.no/). For projects registered in the RiS database and carried out in compliance with the Kings Bay AS, no specific permissions are required for marine work. The work does not include protected or endangered species.

## Supporting information

S1 Fig Midday atmospheric light during Polar Night. Spectral irradiance time series measured north of Rijpfjorden, Svalbard (80° 37.79N 22° 4.14E) on January 15, 2017 over midday period. **(a)** Solar (black) and lunar (red) altitude during measurements. Lunar phase was a waning gibbous moon, full on January 12. **(b)** Ratio of 492 nm (solar/lunar light at sensitivity maximum of krill) [1] and both the 557 nm (green) and 630 nm (red) aurora lines [2]. **(c)** Time series of E_PAR_ (400 to 700 nm; upper panel) and 492 nm, 557 nm, and 630 nm light (lower panel). **(d)** Spectral irradiance at 3 time points during the time series shown in (c); with a running mean and 492 nm peaks (black lines), and aurora lines at 557 nm and 630 nm plotted green and red, respectively. For data, see S1 Data.
(DOCX)

S2 Fig Rhythmic oscillations in krill visual sensitivity. To test whether Arctic krill (*T*. *inermis*) showed rhythmic changes in visual sensitivity, and, in turn, warranted further experiments, we collected an individual krill from Kongsfjorden in January, and immediately prepared it for ERG recording. **(a)** ERG magnitude (red line = 1.75 hours running mean) is plotted in response to a 50-ms flash of 488-nm light at 3.65 × 10^9^ photons cm^−2^ s^−1^. Since this animal was in darkness, subjective solar elevation (negative degrees relative to horizon) is plotted for the collection location. Peaks in ERG response magnitude occurred during the time of subjective night. **(b)** Lomb–Scargle periodogram for ERG data in (a), resulted in a peak period at 20.4 hours. Dashed line represents significance at the ɑ = 0.05 level. For data, see S2 Data. ERG, electroretinogram.
(DOCX)

S1 Table Period analysis of acoustic backscatter data. Period (hours) estimates for Kongsfjorden and Rijpfjorden acoustic data (from Fig 2) are provided for discrete depths throughout the water column for January 2018. Periodicity was calculated by Lomb–Scargle periodogram. Gray shading indicates periods within the circadian range (20–28 hours), showing that circadian cycling is spread across the water column. The full moon occurred on January 2.
(DOCX)

S1 Data Light and bioacoustic data for Figs 1 and 2 and S1 Fig. (XLSX)

S2 Data Electrophysiological and associated data for Figs 3 and 4 and S2 Fig. (XLSX)

## Abbreviations

ADCP: acoustic doppler current profiler
DVM: diel vertical migration
ERG: electroretinogram
LVM: lunar vertical migration
RiS: Research in Svalbard
TSA: Time Series Analysis
