# Supplementary material for: Photophysiological cycles in Arctic krill are entrained by weak midday twilight during the Polar Night
Source: PLoS Biol. 2021 Oct 19;19(10):e3001413. doi: 10.1371/journal.pbio.3001413 (PMC8525745; doi:10.1371/journal.pbio.3001413)
Supplement: S1 Fig — Spectral irradiance time series measured north of Rijpfjorden, Svalbard (80° 37.79N 22° 4.14E) on January 15, 2017 over midday period. (a) Solar (black) and lunar (red) altitude during measurements. Lunar phase was a waning gibbous moon, full on January 12. (b) Ratio of 492 nm (solar/lunar light at sensitivity maximum of krill) [1] and both the 557 nm (green) and 630 nm (red) aurora lines [2]. (c) Time series of EPAR (400 to 700 nm; upper panel) and 492 nm, 557 nm, and 630 nm light (lower panel). (d) Spectral irradiance at 3 time points during the time series shown in (c); with a running mean and 492 nm peaks (black lines), and aurora lines at 557 nm and 630 nm plotted green and red, respectively. For data, see S1 Data. (DOCX) [file pbio.3001413.s001.docx]

*
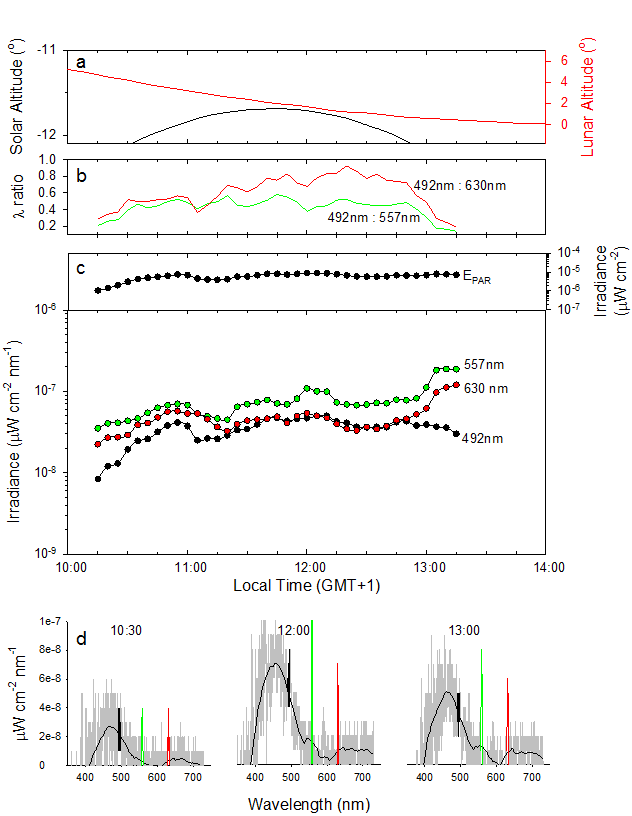
*

**S1 Fig. Mid-day atmospheric light during Polar Night.** Spectral irradiance time series measured north of Rijpfjorden, Svalbard (80º 37.79N 22º 4.14E) on January 15, 2017 over midday period**.** (**a**) Solar (black) and lunar (red) altitude during measurements. Lunar phase was a waning gibbous moon, full on January 12. (**b**) Ratio of 492 nm (solar/lunar light at sensitivity maximum of krill) [[1]](https://paperpile.com/c/UgRDx0/M3GtL) and both the 557 nm (green) and 630 nm (red) aurora lines [[2]](https://paperpile.com/c/UgRDx0/JfvGG). (**c**) Time series of E_PAR_ (400-700 nm; upper panel) and 492 nm, 557 nm, and 630 nm light (lower panel). (**d**) Spectral irradiance at three time points during the time series shown in (c); with a running mean and 492 nm peaks (black lines), and aurora lines at 557 nm and 630 nm plotted green and red, respectively. For data, see S1 Data.

References

1. [Cohen JH, Berge J, Moline MA, Sørensen AJ, Last K, Falk-Petersen S, et al. Is Ambient Light during the High Arctic Polar Night Sufficient to Act as a Visual Cue for Zooplankton? PLoS One. 2015;10: e0126247.](http://paperpile.com/b/UgRDx0/M3GtL)
2. [McLennan JC, Shrum GM. On the origin of the auroral green line 5577 Å, and other spectra associated with aurora borealis. Proc R Soc A. 1925;108: 501–512.](http://paperpile.com/b/UgRDx0/JfvGG)
