# Supplementary material for: Photophysiological cycles in Arctic krill are entrained by weak midday twilight during the Polar Night
Source: PLoS Biol. 2021 Oct 19;19(10):e3001413. doi: 10.1371/journal.pbio.3001413 (PMC8525745; doi:10.1371/journal.pbio.3001413)
Supplement: S2 Fig — To test whether Arctic krill (T. inermis) showed rhythmic changes in visual sensitivity, and, in turn, warranted further experiments, we collected an individual krill from Kongsfjorden in January, and immediately prepared it for ERG recording. (a) ERG magnitude (red line = 1.75 hours running mean) is plotted in response to a 50-ms flash of 488-nm light at 3.65 × 10^9^ photons cm−2 s−1. Since this animal was in darkness, subjective solar elevation (negative degrees relative to horizon) is plotted for the collection location. Peaks in ERG response magnitude occurred during the time of subjective night. (b) Lomb–Scargle periodogram for ERG data in (a), resulted in a peak period at 20.4 hours. Dashed line represents significance at the ɑ = 0.05 level. For data, see S2 Data. ERG, electroretinogram. (DOCX) [file pbio.3001413.s002.docx]

**
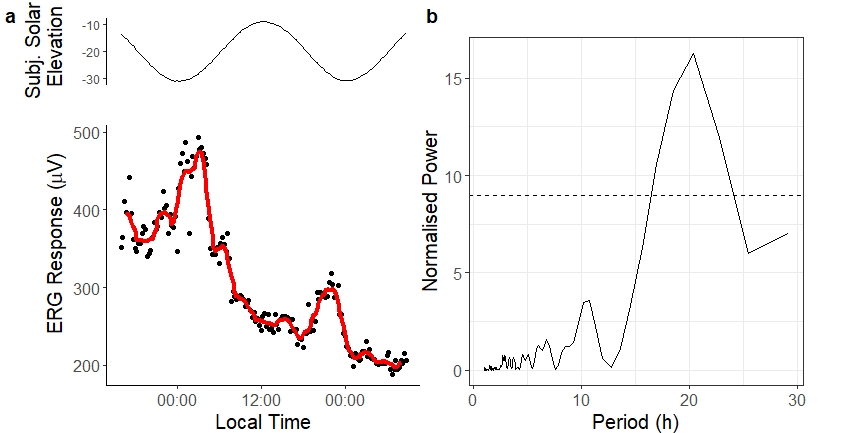
**

**S2 Fig.** **Rhythmic oscillations in krill visual sensitivity.**  To test whether Arctic krill (*T. inermis*) showed rhythmic changes in visual sensitivity, and in turn warranted further experiments, we collected an individual krill from Kongsfjorden in January, and immediately prepared it for electroretinogram (ERG) recording. (**a**) ERG magnitude (red line = 1.75 h running mean) is plotted in response to a 50 ms flash of 488 nm light at 3.65x10^9^ photons cm^-2^ s^-1^. Since this animal was in darkness, subjective solar elevation (negative degrees relative to horizon) is plotted for the collection location. Peaks in ERG response magnitude occurred during the time of subjective night. (**b**) Lomb-Scargle periodogram for ERG data in (a), resulted in a peak period at 20.4 h. Dashed line represents significance at the ɑ = 0.05 level. For data, see S2 Data.
