# Supplementary material for: Photophysiological cycles in Arctic krill are entrained by weak midday twilight during the Polar Night
Source: PLoS Biol. 2021 Oct 19;19(10):e3001413. doi: 10.1371/journal.pbio.3001413 (PMC8525745; doi:10.1371/journal.pbio.3001413)
Supplement: S1 Table — Period (hours) estimates for Kongsfjorden and Rijpfjorden acoustic data (from Fig 2) are provided for discrete depths throughout the water column for January 2018. Periodicity was calculated by Lomb–Scargle periodogram. Gray shading indicates periods within the circadian range (20–28 hours), showing that circadian cycling is spread across the water column. The full moon occurred on January 2. (DOCX) [file pbio.3001413.s003.docx]

**S1 Table.**  **Period** **analysis of acoustic backscatter data.** Period (hours) estimates for Kongsfjorden and Rijpfjorden acoustic data (from Fig. 2) are provided for discrete depths throughout the water column for January 2018. Periodicity was calculated by Lomb-Scargle periodogram. Grey shading indicates periods within the circadian range (20-28 h), showing that circadian cycling is spread across the water column. The full moon occurred on January 2.

|  | **Kongsfjorden [Period]** | | | | **Rijpfjorden [Period]** | | | |
| --- | --- | --- | --- | --- | --- | --- | --- | --- |
| Depth | *Week 1: Jan 01- Jan 08* | *Week 2: Jan 09- Jan 16* | *Week 3: Jan 17 - Jan 24* | *Week 4: Jan 25 - Jan 31* | *Week 1: Jan 01- Jan 08* | *Week 2: Jan 09- Jan 16* | *Week 3: Jan 17 - Jan 24* | *Week 4: Jan 25 - Jan 31* |
| 32 | 13.7 | 23.1 | 25.4 | 12.0 | 27.7 | 32.1 | 25.4 | 25.0 |
| 52 | 27.2 | 30.0 | 21.4 | 20.5 | 26.1 | 36.0 | 24.3 | 36.0 |
| 72 | 21.9 | 23.2 | 23.4 | 12.1 | 27.3 | 25.4 | 7.89 | 13.2 |
| 130 | 28.3 | 18.4 | 24.0 | 20.5 | 23.4 | 25.0 | 22.0 | 25.4 |
| 149 | 15.3 | 30.0 | 24.3 | 21.7 | 21.7 | 25.4 | 31.6 | 30.5 |
| 170 | 27.4 | 23.4 | 33.3 | 11.7 | 21.2 | 33.3 | 9.73 | 24.0 |
| 190 | 26.8 | 12.0 | 14.5 | 24.0 | 34.6 | 15.5 | 25.7 | 23.7 |
